# 我国胸外科发展趋势与分会的任务

**DOI:** 10.3779/j.issn.1009-3419.2014.07.01

**Published:** 2014-07-20

**Authors:** 天佑 王

**Affiliations:** 100050 北京，首都医科大学附属北京友谊医院 Beijing Friendship Hospital, Capital Medical University, Beijing 100050, China

进入21世纪，外科学开始了一个革命性飞跃的发展阶段。许多外科新理念，如微创外科理念，精准外科理念，快速康复外科理念等在胸外科得到了广泛的体现和应用。现代高科技的成果，如微创外科技术、遥控遥测计算机技术、分子生物学、基因检测和治疗技术等，逐步在胸外科得到引入、转化和应用。特别是以电视胸腔镜技术为代表的微创胸外科的迅速发展和推广，逐步取代传统胸外科技术，使胸外科正在经历一场传统胸外科向现代精准微创胸外科转变的深刻革命。

据不完全统计，我国现有胸外科医师（包括同时做其他专科如心外科等）约6, 000余人，每年胸外科手术超过30万例，中国大陆胸外科年手术量超过1, 000例大型医疗中心约51个（2012年），本次年会注册的胸外科医师分会会员超过3, 200人。我国胸外科的总体规模，胸外科医师队伍，在全世界名列前茅，甚至可能居于首位，其技术水平也在飞速发展，正在或者已经与世界接轨。

## 发展趋势

### 一 胸外科手术量继续逐年增加

统计中国大陆10个大型医疗中心胸外科近五年来（2008年-2012年）年手术量增加68%（17, 358例-29, 059例），不少肿瘤医院的胸外科年手术量5年来增长超过1倍。目前这种增长趋势仍在持续，甚至更为明显。如上海肺科医院上月手术量超过560例，有望今年手术量超过6, 000例（2012年为4, 200例）。现阶段我国胸外科手术量持续增长的原因考虑如下：

1.胸部肿瘤（肺癌、食管癌等）发病率增加，发病患者数增加在未来五年内逐渐体现出来，环境污染如果不能得到有效遏制，肺癌的发病患者数只会上升而不会下降。

2.医疗保障制度的继续落实，使农村患者的经济支付能力提高。

3.医疗保健观念提高，定期体检数量加大，胸外科疾病早期诊断和发现的数量增大。

4.其他，如医院管理者的市场竞争意识的增强等。

### 二 电视胸腔镜技术为代表的微创胸外科将进一步推广和覆盖，将成为胸外科的主要手术方式

笔者统计2012年国内14个大型医学中心电视辅助胸腔镜手术（video-assisted thoracic surgery, VATS）占全部胸外科手术的比例为25%-65%。目前增长势头很猛，80%-90%的手术均可在胸腔镜下进行。刘伦旭教授完成了全胸腔镜下双袖状肺叶切除术、张逊教授首创创新的方法胸腔镜手术治疗食管癌巨大憩室。我国的VATS已具一定规模和水平。目前我国有12台达芬奇机器人设备，多家医院应用机器人进行了胸外科手术，获得良好效果。我分会微创胸外科专家委员会，对微创外科发展起到了重要作用。我会特别注重青年胸外科医师的胸腔镜技术的培训，连续两年组织这项技术的菁英赛，以推广胸腔镜手术技术。未来五年中，三维电视胸腔镜技术将逐步推开，将简化和降低手术难度，缩短技术培训曲线。经自然腔道如经口食管镜的微创技术将在胸外科进一步探索和应用。达芬奇机器人技术有望进一步推广。在未来的五年中，VATS和机器人手术将在中国有一个较大的发展，上一个新的台阶，微创胸外科手术将成为胸外科的主要手术方式，传统胸外科手术技术逐步转变为补充术式。

### 三 肺癌外科技术的新动向

1.局限性肺切除是否能成为一部分肺癌的标准术式？局限性肺切除的手术方法如何改进？是目前肺癌外科正在探讨的新问题。由于低剂量螺旋计算机断层扫描（computed tomography, CT）筛查的推广应用，Ia期肺癌或更小的肺孤立结节早期发现明显增多，临床X线表现为肺部磨玻璃影（ground-glass opacity, GGO）病例也明显增多，另外生长缓慢的高分化腺癌似乎也在增多，这些病例是否需行肺叶切除？目前正在进行多中心、大样本的临床研究。

肺段切除和肺楔形切除以及精准切除术（非典型局限性肺切除）何种术式较好？也需要进一步临床研究。关于肺部小结节的定位（CT定位穿刺、电磁导航定位活检、术中定位等）、术前病理诊断、准确切除都需要进一步研究。

2.双肺多发结节或病变的术前准确诊断和治疗方法选择，需进一步研究。

3.基于分子生物学和基因测定的肺癌外科手术技术初露端倪，需深入研究。

4.靶向治疗在肺癌的综合性治疗的地位更为重要，将会与外科手术作为新的组合方式，成为早期肺癌和中晚期肺癌的综合治疗新的模式，并明显提高治疗效果。

### 四 食管癌外科治疗动向

1.右侧开胸手术正在推广应用，由于便于清扫纵隔淋巴结，一定程度上提高5年生存率，有代替左侧开胸的趋势。

2.食管癌手术范围大，创伤大，手术时间长，进行VATS比肺VATS发展缓慢，手术方式繁多，各家经验不同。有胸腔镜手术、开腹+胸腔镜手术、附加颈部切口手术等等。随着开展例数的增加和经验积累，逐步达到共识和推广。

### 五 肺移植术有待于进一步发展

肺移植总数减少，医院集中为目前肺移植发展趋势。2012年，国内有肺移植手术资质者为26家医院，总数为213例，与国际水平有较大差距，需进一步发展。

### 六 其他，如纵隔外科、快速康复胸外科等技术有望在未来数年中获得较大进展

## 分会的任务

中国医师协会胸外科医师分会是根据国家相关法律依法成立的中国注册胸外科医师的唯一的全国性行业协会，是我国胸外科医师自愿组成的、带有学术性的全国性行业组织，是中国医师协会的二级机构。

本会的宗旨是发挥行业指导、服务、自律、协调、监督、管理作用，团结和组织全国胸外科医师遵守国家宪法法律，法规和政策，弘扬以人为本、救死扶伤的人道主义职业道德，维护医师合法权益，努力提高医疗水平和服务质量，为全国人民的健康和社会主义服务。

我会成立六年来，在中国医师协会的领导下，按照以上宗旨做了大量工作，由小到大，有了很大的发展，其影响力也逐步扩大。新的第三届委员会已经成立，去年我曾建议的大力发展会员和改革组织机构俩项任务完成较好，现对今后一届的其他任务建议如下，以供参考。

我想，制定分会任务的主要指导思想为：①努力促进和引导我国胸外科事业的创新和发展；②践行协会制定的行业指导、服务、自律、协调、监督和管理等方针，团结广大胸外科医师，把分会办成全国胸外科医师之家；③在中国胸外科医师协会的领导下，努力完成政府安排的各项工作，成为政府的得力助手。分会任务建议如下：

1.建立CATS数据库。长期以来，我们拿不出全国胸外科的准确数据，只是根据政府行政部门的统计估计一些数据，不详尽、不科学，急需建立我会的数据库。当然对于这样一个庞大国家和当前的社会状况来讲，这是一件十分困难的事，但是再困难也必须建立。

2.积极参与和做好医师定期考核工作，争取逐步获得胸外科医师专科医师准入资格认证、定期考评工作的职能。专科医师的准入认证目前由政府掌握，卫生部将逐步下放一些工作由协会来做，医师定期考核就是一例，我们一定要做好这项工作，争取政府能下放更多的职能交给协会来做。

3.开好年会。年会是分会重要工作，内容分为两部分：①协会与人文交流；②胸外科学术大会。近年来，人文部分有所发展，应继续保持，学术交流部分进步较小。根据国内情况，需要进一步加强学术交流部分的工作，应发挥各专家委员会的作用，把胸外科学术部分扩大，实属必要，可设立更多的分会场进行交流。

4.办好分会通讯，创办分会的机关刊物。目前我会开始内部出版（通讯）和*Innovation*杂志（与心血管外科医师分会合办的杂志，正在试刊）。分会将努力争取创办自己的机关刊物胸外科杂志，请大家多提意见和建议，争取自己的刊物早日与大家见面。

5.加强对外交流。协会应派遣代表团参加国际会议，参加会议举办的各种活动。与国外协会建立合作关系，互换会员。把只邀请国外专家来我会讲学，逐步改变为双向式。推荐我会的专家到国外讲学，介绍我国胸外科的发展，展示我们自己的成果。不久的将来，中国医师协会胸外科医师分会必将自立于世界学术之林，实现中国梦。

